# *Osbpl2* deficiency inhibits Rho/ROCK2/p-ERM signaling and impairs actin cytoskeletal regulation in auditory cells

**DOI:** 10.7555/JBR.38.20240389

**Published:** 2025-05-20

**Authors:** Cheng Zhang, Qian Yang, Yajie Lu, Qinjun Wei, Rong Zhou, Guangqian Xing, Xin Cao, Zhibin Chen, Jun Yao

**Affiliations:** 1 Department of Medical Genetics, School of Basic Medical Sciences, Nanjing Medical University, Nanjing, Jiangsu 211166, China; 2 Jiangsu Key Laboratory of Xenotransplantation, Nanjing Medical University, Nanjing, Jiangsu 211166, China; 3 Department of Physiology, School of Basic Medical Sciences, Nanjing Medical University, Nanjing, Jiangsu 211166, China; 4 Department of Otolaryngology, the First Affiliated Hospital of Nanjing Medical University, Nanjing, Jiangsu 210029, China; 5 Department of Otolaryngology-Head and Neck Surgery, the Affiliated Taizhou People's Hospital of Nanjing Medical University, Taizhou School of Clinical Medicine, Nanjing Medical University, Taizhou, Jiangsu 225300, China

**Keywords:** *OSBPL2*, hearing loss, actin cytoskeleton, Rho/ROCK signaling, p-ERM

## Abstract

A mutation in oxysterol-binding protein-like 2 ( *OSBPL2*) has been identified as the genetic cause of autosomal dominant nonsyndromic hearing loss (DFNA67, Online Mendelian Inheritance in Man No. 616340). However, the pathogenesis of the *OSBPL2* mutation in DFNA remains unclear. Our previous work showed that *Osbpl2* deficiency impaired cell adhesion in auditory HEI-OC1 cells. In addition, loss of hair cells (HCs) and morphological abnormalities of HC stereocilia were detected in *OSBPL2*-knockout pigs, suggesting that OSBPL2 plays an important role in regulating the actin cytoskeleton in auditory cells. In the present study, we found that *Osbpl2* deficiency inhibited the Rho/ROCK2 signaling pathway and downregulated phosphorylated ezrin-radixin-moesin (p-ERM), resulting in abnormal F-actin morphology in HEI-OC1 cells and stereociliary defects in mouse HCs. The present study demonstrates the underlying mechanism of OSBPL2 in regulating the actin cytoskeleton in HCs, contributing to a deeper understanding of the pathogenesis of *OSBPL2* mutations in DFNA.

## Introduction

Hearing loss is one of the most common sensory disorders, affecting over 5% of the world's population ^[ 1] ^. Hereditary hearing loss accounts for approximately half of all cases of congenital sensorineural hearing loss, which may be caused by genetic mutations or by the combined effects of genetic and environmental factors ^[ 2] ^. Because of the unique physiology and structure of the inner ear, the auditory function is very sensitive to mutations in genetic loci. It is known that the proteins encoded by the deafness-related genes are implicated in multiple functions in the ear, such as cochlear fluid homeostasis, gap junctions, the cytoskeleton of hair cells (HCs), ion channels, synaptic transmission, intracellular transport, and stereocilia morphology and function ^[ 3– 5] ^. Mutations in these genes lead to malfunctions of the inner ear and eventually to auditory disorders.

Actin is a key cytoskeletal component that forms specific actin-rich structures in sensory HCs, including the cuticular plate, an actin meshwork that anchors stereocilia; the lateral wall, which features an actin-spectrin lattice; and stereocilia, which are arranged as a tightly bundled array on HCs' apical membrane and are essential for mechanoelectrical signal transduction ^[ 6] ^. Sensory HCs, including three rows of outer HCs (OHCs) and one row of inner HCs (IHCs), detect sound through the deflection of stereocilia and transduce auditory stimuli into electrical impulses ^[ 7] ^. The development and maintenance of stereocilia rely on the precise regulation of actin filament (F-actin) assembly. Deafness-causing mutations that result in defects in the cytoskeletal organization or dimensions of the HC stereocilia have been mapped to genes encoding actin or actin regulators, such as *DIAPH1* (DFNA1), *MYO7A* (DFNA11), *ACTG1* (DFNA20/26), *RDX* (DFNB24), *TRIOBP* (DFNB28), *ESPN* (DFNB36), *PJVK* (DFNB59), *TPRN* (DFNB79), and *SMPX* (DFN4) ^[ 8– 13] ^. Additionally, an increasing number of deaf mutant mice with malformed stereocilia highlight the importance of stereociliary morphogenesis for the function of HCs as mechanosensory receptors ^[ 14– 15] ^.

Oxysterol-binding protein-like 2 (OSBPL2), a member of the oxysterol-binding protein (OSBP)-related protein (OSBP/ORPs) family, plays critical roles in regulating lipid sensing and transport, signal transduction, endocytosis, autophagy, and actin-dependent cell dynamics ^[ 16– 18] ^. We previously identified a novel *OSBPL2* mutation associated with autosomal dominant nonsyndromic hearing loss (DFNA67, Online Mendelian Inheritance in Man [OMIM] No. 606731) in a large affected Chinese family ^[ 19] ^. Subsequent studies have confirmed the implication of *OSBPL2* mutations in DFNA among German and Mongolian populations ^[ 20– 21] ^. However, the pathogenic mechanisms of *OSBPL2* mutations in DFNA remain unclear. OSBPL2 localizes to HC stereocilia ^[ 20] ^. Previously, we found that allelic mutations in porcine *OSBPL2* led to progressive hearing loss with similar pathogenicity to that in humans, and that pathological changes in HC stereocilia were also detected in *OSBPL2*-knockout pigs ^[ 22] ^. Additionally, *Osbpl2* deficiency disrupts phosphoinositide homeostasis in the cilia, causing kinocilia/cilia defects, abnormal cochlear development, and abnormal Sonic Hedgehog (Shh) signal transduction in auditory cells ^[ 23] ^. Notably, OSBPL2 regulates the Ras homolog (Rho) signaling pathway, which affects the actin cytoskeleton, thereby affecting hepatocellular migration, adhesion, and proliferation ^[ 24] ^. Whether OSBPL2 has a similar effect on auditory cells remains to be further investigated.

In the present study, we used *Osbpl2*-knockout (KO) HEI-OC1 cells and mouse models to investigate the stereocilia-associated mechanisms of *Osbpl2* deficiency.

## Materials and methods

### Antibodies and reagents

The antibodies used in the current study included anti-OSBPL2 (1∶200; Cat. #14751-1-AP, Proteintech, Rosemont, IL, USA), monoclonal rabbit anti-ROCK1 (1∶500; Cat. #A11158, Abclonal, Wuhan, China), monoclonal rabbit anti-ROCK2 (1∶500; Cat. #A2395, Abclonal), polyclonal rabbit anti-ERM (1∶1000; Cat. #A21093, Abclonal), polyclonal rabbit anti-p-ERM (1∶1000; Cat. #AP1594, Abclonal), anti-GAPDH (1∶5000; Cat. #5174, Cell Signaling Technology, Danvers, MA, USA), donkey anti-rabbit IgG (H+L) Alexa Fluor 546 (1∶500; Cat. #A10040, Invitrogen, Carlsbad, CA, USA), peroxidase-conjugated goat anti-rabbit (1∶5000; Cat. #PR30011, Proteintech), and peroxidase-conjugated goat anti-mouse (1∶5000; Cat. #PR30012, Proteintech).

The reagents used in the current study included Rho GTPase activator (lysophosphatidic acid, LPA; Cat. #HY-107614, MedChem Express, Monmouth Junction, NJ, USA), Rho GTPase inhibitor fasudil-HCl (FSDL; Cat. #HA-1077, Selleck, Houston, TX, USA), and DAPI (Cat. #F6057, Sigma-Aldrich, St. Louis, MO, USA).

### Cell culture and treatment

The *Osbpl2*-KO cell line was constructed and used as previously described ^[ 25] ^. Wild-type (WT) and *Osbpl2*-KO HEI-OC1 cells were cultured in Dulbecco's modified Eagle's medium (DMEM; Cat. #C11995500BT, Gibco, Grand Island, NY, USA) with 10% fetal bovine serum (FBS; Cat. #10099141, Gibco) at 33 ℃ under 10% CO _2_. Meanwhile, *Osbpl2*-KO and WT HEI-OC1 cells were treated with LPA or FSDL at various concentrations.

### Animals

*Osbpl2*-KO mice on a C57BL/6J background were produced by Cyagen Biosciences (Suzhou, China) and propagated in the Animal Core Facility of Nanjing Medical University. All mice used in the experiments were maintained under the same conditions as previously reported ^[ 23]^ and were given *ad libitum* access to fresh water and a rodent diet. All animal experiments were approved by the Institutional Animal Care and Use Committee of Nanjing Medical University (Approval No. IACUC-1801003). All efforts were made to minimize the number of animals used and to prevent suffering.

### RNA-sequencing (RNA-seq) analysis

Total RNA from *Osbpl2*-KO and WT HEI-OC1 cells was isolated using TRIzol reagent (Invitrogen) according to the manufacturer's instructions. The cDNA libraries were constructed and sequenced by Applied Protein Technology (Shanghai, China). RNA-seq was performed on the Illumina HiSeq 2500 system according to the manufacturer's protocols. After raw read filtering and sequencing error-rate checking (Q20 and Q30 > 90%), the high-quality, clean paired-end reads with no GC bias were mapped to the reference genome using HISAT2 (v.2.1.0). The values of fragments per kilobase of transcript per million mapped reads (FPKM) were calculated for each annotated gene. Differentially expressed genes (DEGs) were identified using the R-package DESeq2 (v3.8) with |log _2_(fold change [FC])| > 1.0, *Q*-value < 0.01, and false discovery rate (FDR) < 0.01. Transcriptional profiles of *Osbpl2*-KO and WT samples were compared using Cuffdiff. Principal component analysis was performed to assess correlations within the RNA-seq datasets. DEGs were analyzed using Subread v2.06 with the featureCounts package ( https://subread.sourceforge.net/) and were mapped to Gene Ontology (GO) terms ( http://www.geneontology.org/). Functional enrichment was analyzed using the ClusterProfiler R package (v4.2.2), and the Kyoto Encyclopedia of Genes and Genomes (KEGG) pathways enriched in DEGs were identified according to the enrichment threshold value (|log _2_FC| > 1.0, *P* < 0.05).

### Quantitative reverse transcription-PCR (RT-qPCR)

The mouse cochleae were collected from 6-month-old *Osbpl2*-KO and WT mice (six mice per genotype). Total RNA was extracted from mouse cochleae using TRIzol reagent (Invitrogen). One microgram of total RNA was used for cDNA synthesis using a HiScript Ⅱ One Step qRT-PCR Kit (Vazyme, Nanjing, China). qPCR was performed on a StepOne Plus system (Applied Biosystems, Foster City, CA, USA) with ChamQ SYBR qPCR Master Mix (Vazyme). The comparative Ct method (2 ^−ΔΔCT^) was applied to evaluate the relative mRNA expression levels of the selected genes. Each qRT-PCR analysis was performed in triplicate, and *Gapdh* served as an internal control for consistent comparison ( ***Supplementary Table 1***, available online).

### Auditory test

Auditory function was assessed using the auditory brainstem response (ABR) test as previously described ^[ 23] ^. Briefly, mice were anesthetized by intraperitoneal injection of xylazine (10 mg/kg) and placed in a soundproof chamber. Acoustic stimuli, including click and tone-burst stimuli (4, 8, 16, 24, and 32 kHz), were generated by the TDT system (Tucker Davis Technologies, Alachua, FL, USA). The ABR waveforms of evoked potentials were recorded at varying stimulus intensities with 5 dB sound pressure level intervals and visualized using BioSigRZ software (Update: March 2024). The ABR threshold was determined as the lowest stimulus intensity that induced a reproducible ABR waveform and was analyzed in triplicate at each frequency.

### Immunoblotting assay

Mouse tissues and HEI-OC1 cells were collected and homogenized in ice-cold RIPA buffer (Beyotime, Shanghai, China) containing a protease and phosphatase inhibitor cocktail (MedChem Express) for 30 min. After centrifugation at 4 ℃ (12000 *g*, 10 min), the proteins in the supernatant were separated by 10% or 12% SDS-PAGE gels and transferred to polyvinylidene difluoride membranes using the Trans-Blot Turbo transfer system (Bio-Rad, Hercules, CA, USA). After blocking with 5% nonfat dried milk in Tris-buffered saline containing 0.05% Tween-20 (pH 7.4) at room temperature for one hour, the membrane was incubated with diluted primary antibodies at 4 ℃ overnight. The membrane was then incubated with a horseradish peroxidase-conjugated secondary antibody at room temperature for one hour. The ECL chemiluminescence kit (Abclonal) was used to visualize the detected proteins.

### Immunofluorescence (IF) staining

The mouse cochlea was carefully removed from the skull and dissected from the temporal bone using a method similar to that previously described ^[ 23] ^. The dissected cochleae were fixed in 4% paraformaldehyde in phosphate-buffered saline (PBS, pH 7.4) at 4 ℃ overnight and then decalcified using 10% ethylenediaminetetraacetic acid for three to four days. After the removal of covering bone from the cochlea, the basilar membrane was dissected from the modiolus around the inner edge of the spiral lamina, permeabilized, and blocked using 0.1% Triton X-100 containing 10% normal goat serum. The basilar membrane was then incubated with the primary antibodies at 4 ℃ overnight, rinsed, and incubated with Alexa Fluor 546-conjugated secondary antibodies or phalloidin (1∶500, Cat. #A10040, Invitrogen) or phalloidin (1∶200, Cat. #40774ES03, Yeasen, Shanghai, China) at room temperature for one hour. Regions ≥ 200 μm from different turns of mouse cochleae were photographed using a laser scanning confocal microscope (Carl Zeiss LSM 700, Oberkochen, Germany). The intact HCs were manually counted using ImageJ software (v1.52). A minimum of three independent cochleae were collected from each experimental group and used for each experiment.

HEI-OC1 cells were cultured on sterilized coverslips and fixed in 4% paraformaldehyde at room temperature for 15 min. The fixed cells were permeabilized with 0.1% Triton X-100, blocked in PBS containing 5% donkey serum, and incubated with phalloidin at room temperature for one hour. The cell nuclei were then counterstained using DAPI. Images were captured using a laser scanning confocal microscope (Carl Zeiss) and analyzed using ZEISS ZEN 3.2 software.

### Scanning electron microscopy (SEM)

The morphology and alignment of HCs on the basilar membrane were examined by SEM. Briefly, the dissected cochleae were fixed using 5% glutaraldehyde in sodium cacodylate buffer (0.1 mol/L, pH 7.2) overnight and decalcified using 10% ethylenediaminetetraacetic acid for three to four days. The basilar membrane was dissected as mentioned above and dehydrated in a gradient ethanol solution. The dehydrated samples were mounted on aluminum specimen stubs, sputter-coated with gold particles, and observed by SEM (Hitachi S-800, Japan). The HC orientation was measured by the angle formed by the V-shaped stereociliary bundle and its deviation from the mediolateral axis of the epithelium using ImageJ software (v1.52), and the data were plotted in rose diagrams.

### Cochlear explants

Mouse cochleae were dissected from postnatal day three (P3) mice and cultured as previously reported ^[ 26] ^. Briefly, the isolated mouse cochleae were transferred onto poly-L-lysine-coated (Sigma-Aldrich) slides and cultured in DMEM/high glucose medium (Gibco) with 10% fetal calf serum (Gibco) for 24 h. After attachment, cochlear explants were washed twice with PBS and cultured in 2 mL serum-free DMEM/high glucose and F12 medium supplemented with 1% N2 (Invitrogen) and 2% B27 (Invitrogen). Meanwhile, cochlear explants were treated with or without LPA or FSDL for 24 h, then washed twice with PBS and cultured in serum-free medium. Half of the medium was replaced every second day. The cochleae were fixed with 4% paraformaldehyde and stained with phalloidin and anti-p-ERM to analyze the stereocilia morphology and count the p-ERM ^+^ HCs. At least 200 μm of different turns were imaged by a laser scanning confocal microscope. The intact HCs were manually counted using ImageJ software (v1.52). The polarity changes of stereocilia were measured by tracing the roots of stereocilia from HCs in *Osbpl2*-KO and WT mice as previously reported ^[ 27] ^. A minimum of three independent cochlear explants were cultured and used for each experiment.

### Statistical analysis

All data are presented as mean ± standard error of the mean. Comparisons between two groups were performed by an independent, two-tailed Student's *t*-test; for multiple groups, one-way analysis of variance (ANOVA) with post hoc test was used. GraphPad Software Prism 9 (CA, USA) was used for plotting. *P <* 0.05 was considered statistically significant.

## Results

### GO and KEGG enrichment analysis

**Figure 1:**
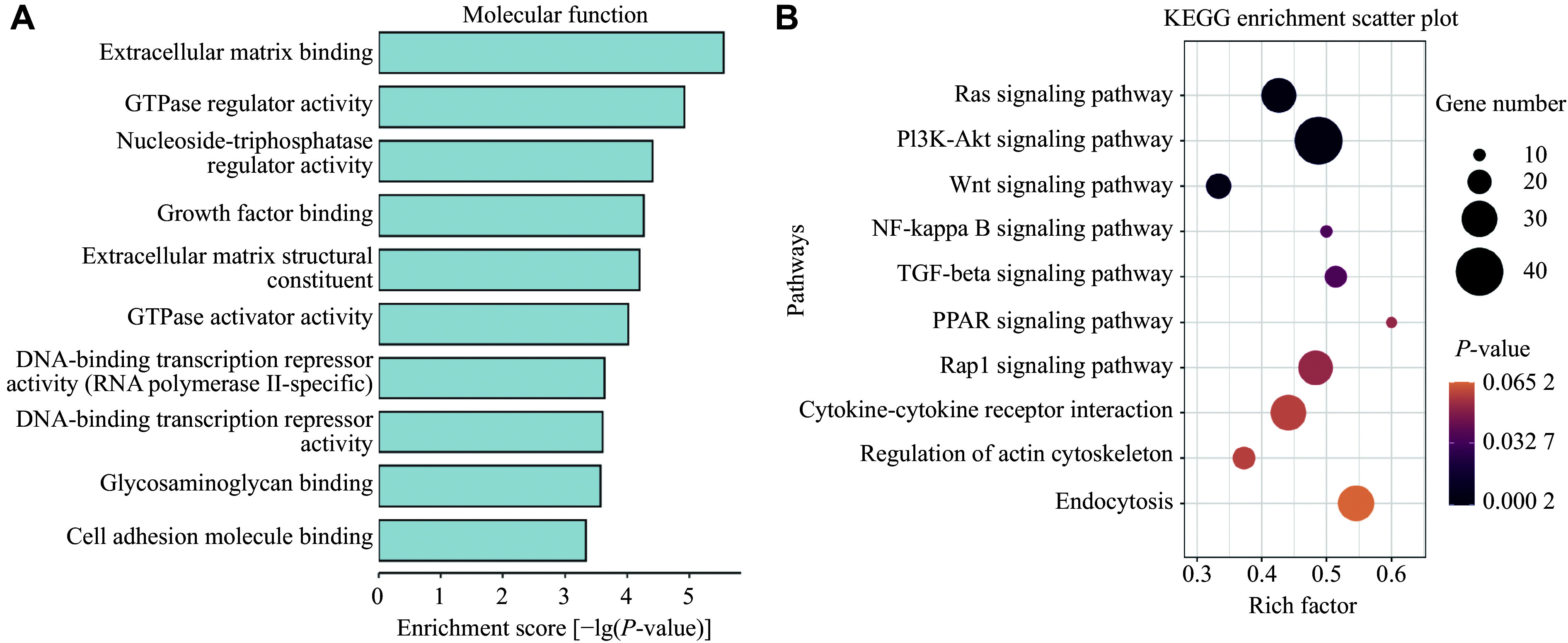
RNA-seq analysis of *Osbpl2*-knockout (KO) and wild-type (WT) HEI-OC1 cells. Gene ontology (A) and KEGG pathway (B) analysis of the differentially expressed genes between *Osbpl2*-KO and WT HEI-OC1 cells ( *P* < 0.05).

RNA-seq was performed to investigate the effect of *Osbpl2* on the expression profile of HEI-OC1 cells. In total, 1093 upregulated and 924 downregulated genes were mapped to the HEI-OC1 reference genome by comparing *Osbpl2*-KO and WT HEI-OC1 cells (|log _2_FC| > 1) ( ***Supplementary Fig. 1***, available online). The GO analysis indicated that molecular functions of OSBPL2 were implicated in extracellular matrix binding, regulation of GTPase activity, nucleoside triphosphatase activity, extracellular matrix structural constituents, and other functions ( ***Fig. 1A***). KEGG analysis indicated that the Ras signaling pathway was one of the significantly enriched in DEGs ( ***Fig. 1B***). Consistently, the key Ras homolog GTPase molecules associated with actin cytoskeleton function, such as Rho family member J ( *RhoJ*), Rho GTPase activating protein 6 ( *Arhgap6*), cytoskeleton-associated protein 5 ( *Ckap5*), and Rho-associated coiled-coil containing protein kinases 1/2 ( *Rock1/2*), significantly differentially expressed between *Osbpl2*-KO and WT HEI-OC1 cells ( ***Supplementary Table 2***, available online). These findings suggest that OSBPL2 may mediate the Rho GTPase signaling pathway and play an important role in the regulation of the actin cytoskeleton in HEI-OC1 cells.

### *Osbpl2* deficiency caused abnormal actin cytoskeleton morphology through the inhibition of Rho GTPase signaling in HEI-OC1 cells

**Figure 2:**
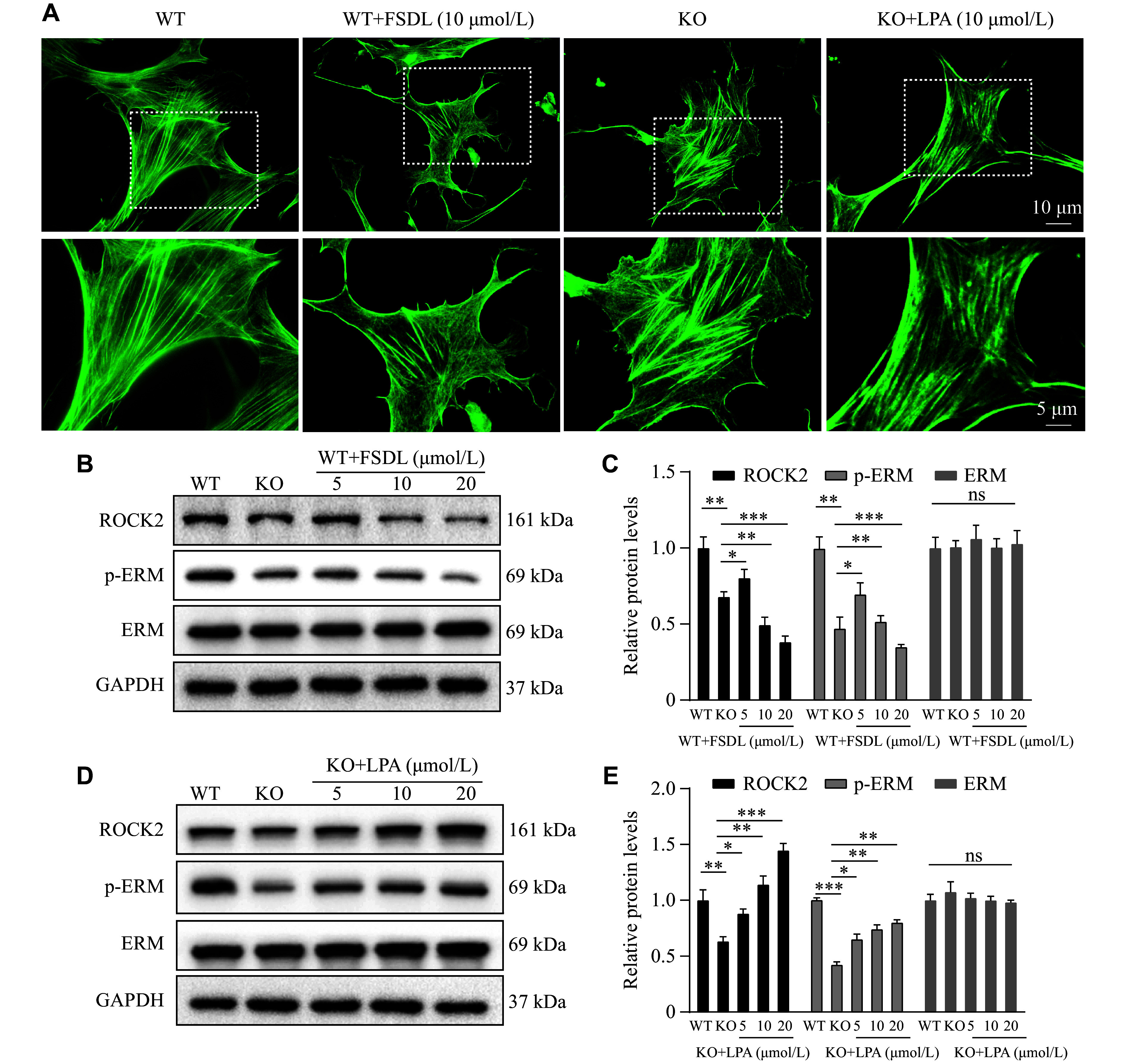
*Osbpl2* deficiency resulted in abnormal morphology of F-actin cytoskeleton in HEI-OC1 cells by inhibiting Rho GTPase signaling. A: Immunofluorescence staining of F-actin cytoskeleton with phalloidin (green) in *Osbpl2*-KO (KO) and wild-type (WT) HEI-OC1 cells treated with or without FSDL or LPA for 24 h at the indicated concentrations. Scale bar: 10 μm. Dashed frames denote the locally zoomed regions as shown below. Scale bar: 5 μm. B–E: Western blotting analysis of ROCK2, ERM, and p-ERM in *Osbpl2*-KO and WT HEI-OC1 cells treated with or without FSDL (B and C) and LPA (D and E) for 24 h at the indicated concentrations. *Osbpl2*-WT HEI-OC1 cells were used as controls. Data are presented as mean and standard error of the mean ( *n* = 3). ^*^ *P* < 0.05, ^**^ *P* < 0.01, and ^***^ *P* < 0.001 by one-way ANOVA analysis. Abbreviations: FSDL, Rho GTPase inhibitor fasudil-HCl; LPA, 1-oleoyl lysophosphatidic acid sodium; ROCK2, Rho-associated coiled-coil-forming kinases 2; ERM, ezrin-radixin-moesin; ns, not significant.

The effect of *Osbpl2* deficiency on the F-actin cytoskeleton in HEI-OC1 cells was investigated by IF staining. Compared with WT controls, F-actin in *Osbpl2*-KO cells was unevenly distributed and disorganized ( ***Fig. 2A***). Meanwhile, *Osbpl2* deficiency led to the downregulation of ROCK2 and p-ERM in *Osbpl2*-KO cells ( ***Fig. 2B***), which was consistent with the abnormal F-actin morphology observed in these cells.

To further explore the molecular mechanisms underlying the effects of *Osbpl2* deficiency on the actin-based cytoskeleton in auditory cells, we treated *Osbpl2*-KO and WT HEI-OC1 cells with the Rho GTPase agonist LPA and the inhibitor FSDL, respectively. The results showed that ROCK2 and p-ERM levels were significantly downregulated in FSDL-treated *Osbpl2*-WT cells ( ***Fig. 2B*** and ***2C***), which exhibited abnormal F-actin morphology similar to that observed in *Osbpl2*-KO cells ( ***Fig. 2A***). However, LPA treatment significantly upregulated ROCK2 and p-ERM levels ( ***Fig. 2D*** and ***2E***) and partially rescued the morphological abnormalities of F-actin in *Osbpl2*-KO cells ( ***Fig. 2A***). These results indicate that *Osbpl2* deficiency inhibits the Rho GTPase signaling pathway, leading to defects in the F-actin cytoskeleton in HEI-OC1 cells.

### *Osbpl2* deficiency caused hearing loss with stereociliary defects

We assessed the auditory function in *Osbpl2*-KO mice by measuring ABR thresholds in response to click and tone-burst stimuli (4, 8, 16, 24, and 32 kHz), and found that these mice at the age of one to six months showed no significant differences in ABR thresholds at low frequencies (4, 8, and 16 kHz) compared with age-matched WT controls. However, they exhibited increased ABR threshold shifts over time at high frequencies (32 kHz; *Supplementary Fig. 2A*, available online). In 6-month-old *Osbpl2*-KO mice, no recognizable ABR waveforms were detected even at a 90 dB sound pressure level stimulus intensity at 32 kHz ( *Supplementary Fig. 2B*, available online). By 12 months of age, *Osbpl2*-KO mice showed significantly elevated ABR threshold shifts at all tested frequencies ( ***Supplementary Fig. 2A***). These results suggest that *Osbpl2*-KO leads to progressive hearing loss in mice, with high-frequency hearing loss appearing early and gradually extending to all frequencies over time. Notably, OSBPL2 was localized to the stereocilia of mouse HCs, as evidenced by IF staining ( ***Supplementary Fig. 3***, available online). *Osbpl2* deficiency caused disorganized and misoriented stereocilia bundles ( ***Supplementary Fig. 4***, available online), thereby resulting in HC polarity disruption. This phenotype first appeared in the basal turn of the organ of Corti in 2-month-old *Osbpl2*-KO mice, corresponding to the onset of high-frequency hearing loss.

**Figure 3:**
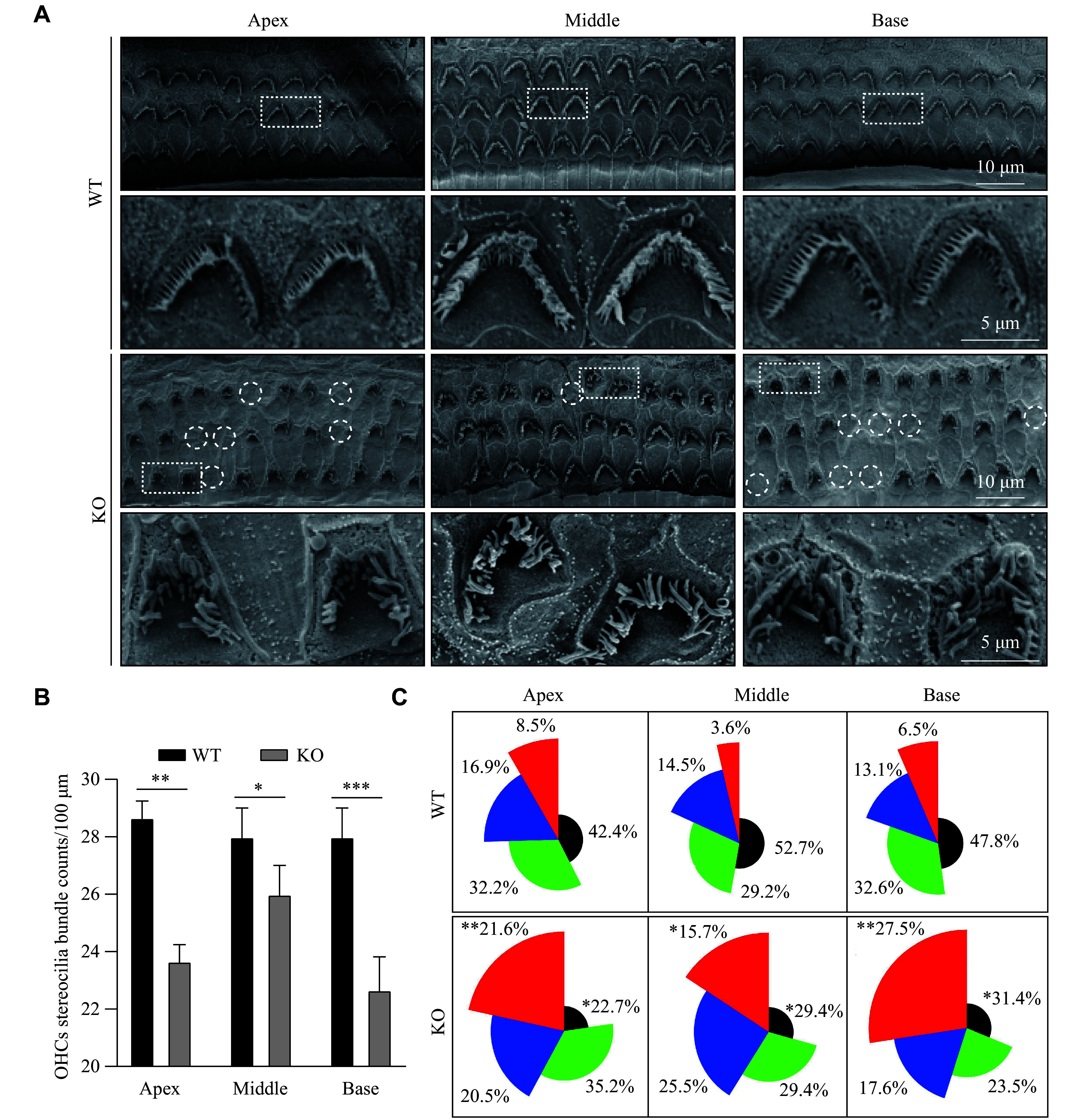
*Osbpl2* deficiency caused OHC stereociliary defects in mouse cochleae. A: Scanning electron microscopy images of HCs at the basal-middle turn of the organ of Corti in 6-month-old *Osbpl2*-KO (KO) mice. White circles denote the regions with a loss of HCs. Scale bar: 10 μm. Dashed frames denote the locally zoomed regions with abnormal stereocilia bundles as shown below. Scale bar: 5 μm. B: Quantification of OHC stereocilia bundles. OHC stereocilia bundles were counted per 100 μm from different turns of the organ of Corti in 6-month-old *Osbpl2*-KO and wild-type (WT) mice (three mice per genotype). C: Distribution of OHC orientation. The angle of deviation between the stereocilia bundle and the mediolateral axis was plotted in rose diagrams, and the pooled data were obtained from 50–100 individual HCs in 6-month-old *Osbpl2*-KO and WT mice (three mice per genotype). Color coding: red, > 30°; blue, 15°–30°; green, 5°–15°; black, < 5°. Data are presented as mean and standard error of the mean. ^*^ *P* < 0.05, ^**^ *P* < 0.01, and ^***^ *P* < 0.001 by two-tailed Student's *t*-test. Abbreviations: HCs, hair cells; OHC, outer hair cell.

To further investigate the effect of *Osbpl2* deficiency on actin-rich stereocilia, we used SEM to examine the ultrastructure of HCs. As shown in ***Fig. 3A*** and ***3B***, *Osbpl2* deficiency led to HC loss and stereociliary abnormalities (including stereocilia loss, absence of top-contact, and abnormal curling and tangling) in 6-month-old *Osbpl2*-KO mice, particularly in the basal turn of the organ of Corti. Based on SEM observations, HC polarity was evaluated by measuring the angle between the stereocilia bundles and the mediolateral axis of the epithelium. The results showed that many actin-rich stereocilia bundles were misoriented in 6-month-old *Osbpl2*-KO mice, especially in the basal turn ( ***Fig. 3C***). These findings indicate that *Osbpl2* deficiency leads to stereociliary defects and HC polarity disruption by affecting the actin cytoskeleton, ultimately resulting in hearing loss.

### *Osbpl2* deficiency inhibited Rho/ROCK2/p-ERM signaling pathway in mouse cochleae

**Figure 4:**
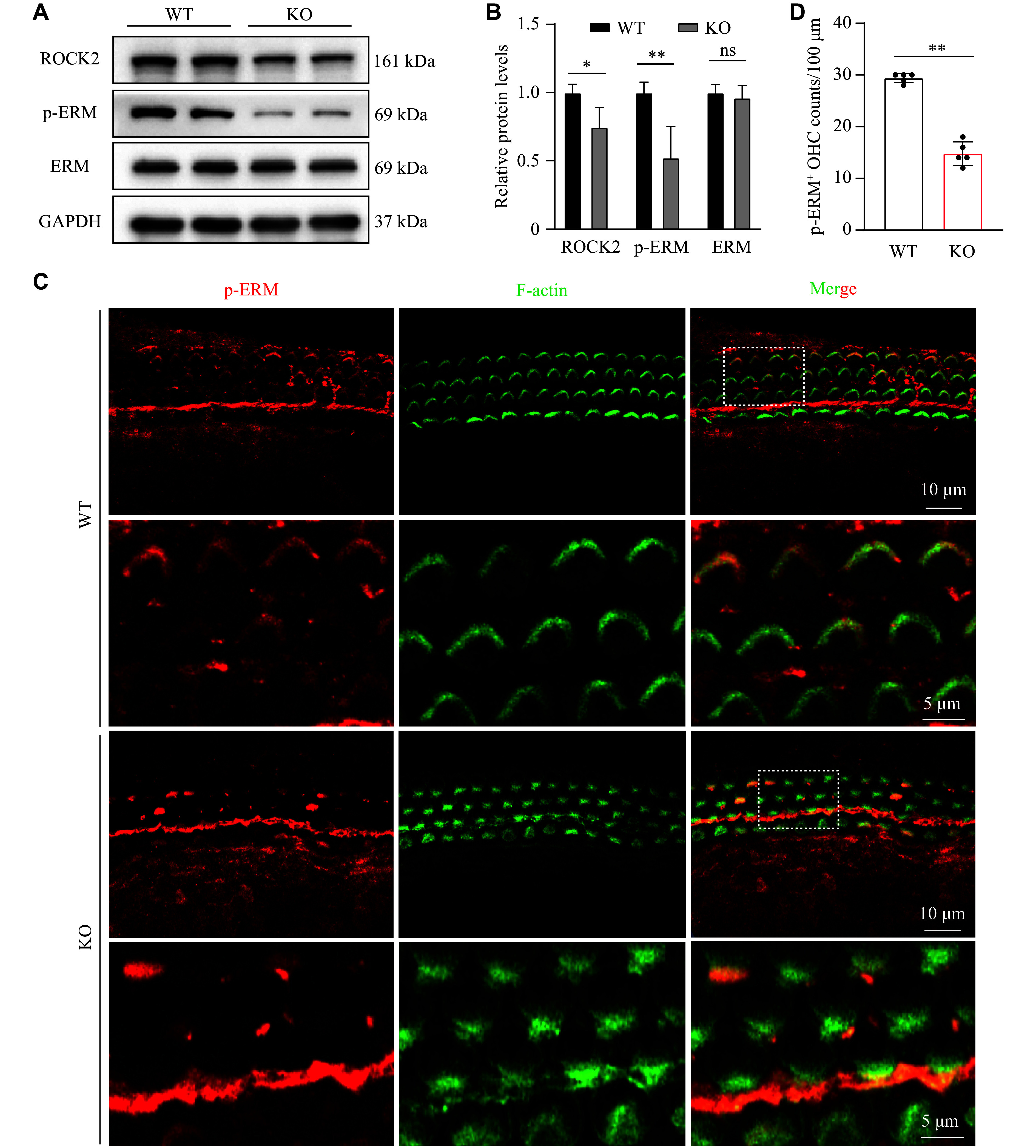
*Osbpl2* deficiency inhibited the Rho/ROCK2/p-ERM signaling pathway in mouse cochleae. A and B: Total protein was extracted from the cochleae of three mice per genotype. The protein levels of ROCK2 and ERM in 6-month-old *Osbpl2*-KO and WT mouse cochleae were detected by Western blotting. B: Quantification of the relative protein levels of ROCK2, p-ERM, and ERM. C: Immunofluorescence staining of the sensory epithelium (basal turn) in 6-month-old *Osbpl2*-KO and WT mice with anti-p-ERM (red) and phalloidin (green). Scale bar: 10 μm. Dashed frames denote the locally zoomed regions with abnormal stereocilia bundles as shown below. Scale bar: 5 μm. D: Quantification of p-ERM ^+^ OHCs. p-ERM ^+^ OHCs were counted per 100 μm from the basal turns of the organ of Corti in 6-month-old *Osbpl2*-KO and WT mice. Data are presented as mean and standard error of the mean ( *n* = 3). ^*^ *P* < 0.05 and ^**^ *P* < 0.01 by two-tailed Student's *t*-test. Abbreviations: ROCK2, Rho-associated coiled-coil-forming kinases 2; ERM, ezrin-radixin-moesin; OHCs, outer hair cells; ns, not significant.

The effects of *Osbpl2* deficiency on HC stereocilia and the F-actin cytoskeleton in HEI-OC1 cells suggest that OSBPL2 is essential for maintaining the actin-based cytoskeleton in auditory cells, likely mediated by the Rho GTPase signaling pathway. Notably, ROCK2, rather than ROCK1, was dominantly expressed in the mouse cochlea ( ***Supplementary Fig. 5***, available online). *Osbpl2* deficiency significantly downregulated the protein levels of ROCK2 in mouse cochleae, consequently inhibiting downstream ERM phosphorylation ( ***Fig. 4A*** and ***4B***; ***Supplementary Fig. 6***, available online). IF staining showed that p-ERM was colocalized with F-actin in V-shaped stereociliary bundles of *Osbpl2*-WT mice, but was significantly decreased in disorganized stereocilia bundles of *Osbpl2*-KO mice ( ***Fig. 4C***). *Osbpl2* deficiency also led to a decrease in p-ERM-positive (p-ERM ^+^) HCs in mouse cochleae ( ***Fig. 4D***). p-ERM is known as a key effector in the transduction of Rho/ROCK2 signaling and is implicated in the maintenance of actin structure in HCs ^[ 10] ^. These results suggest that *Osbpl2* deficiency inhibits the transduction of Rho/ROCK2/p-ERM signaling, leading to stereociliary defects.

**Figure 5:**
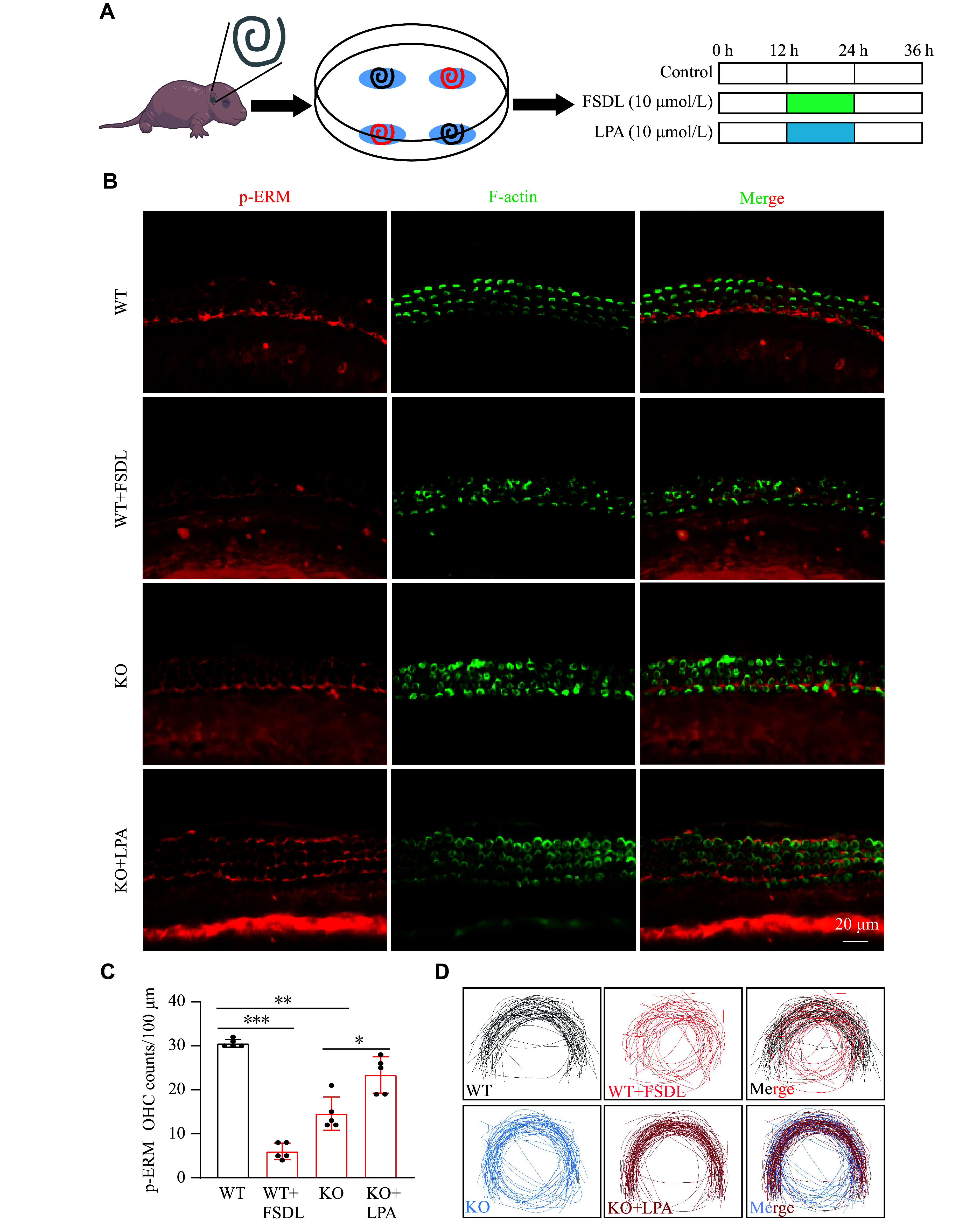
The effects of FSDL and LPA treatment on Rho/ROCK2/p-ERM signaling in *Osbpl2*-KO and WT mouse cochlear explants. A: Schematic diagram of FSDL and LPA treatment of mouse cochlear explants. B: Immunofluorescence staining of the sensory epithelium at the basal turn in *Osbpl2*-KO and WT mouse cochlear explants with anti-p-ERM (red) and phalloidin (green). Scale bar: 20 μm. C: Quantification of p-ERM ^+^ OHCs at the basal turn in *Osbpl2*-KO and WT mouse cochlear explants treated with or without FSDL and LPA. Three independent cochlear explants were cultured and used for each group. One-way ANOVA was used to evaluate the differences in p-ERM ^+^ OHCs among groups. D: OHC stereocilia tracing at the basal turn (100 μm) in *Osbpl2*-KO and WT mouse cochlear explants treated with or without FSDL and LPA. The polarity changes of stereocilia were measured by tracing the roots of OHC stereocilia in *Osbpl2*-KO and WT mouse cochlear explants. Data are presented as mean and standard error of the mean. ^*^ *P* < 0.05, ^**^ *P* < 0.01, and ^***^ *P* < 0.001 by two-tailed Student's *t*-test. Abbreviations: FSDL, Rho GTPase inhibitor fasudil-HCl; LPA, 1-oleoyl lysophosphatidic acid sodium; ERM, ezrin-radixin-moesin; OHCs, outer hair cells.

To further investigate the role of OSBPL2-mediated Rho/ROCK2/p-ERM signaling in maintaining the actin-based stereocilia, we treated mouse cochlear explants with or without LPA/FSDL ( ***Fig. 5A***). Given that the stereociliary defects were initially detected in *Osbpl2*-KO mice exhibiting high-frequency hearing loss, we examined the basal turn of the P3 *Osbpl2*-KO and WT mouse cochlear explants using IF staining. The results showed that the FSDL treatment led to morphological abnormalities in the stereocilia of *Osbpl2*-WT mouse cochlear explants ( ***Fig. 5B***), similar to the defects observed in *Osbpl2*-KO mouse cochlear explants. FSDL treatment also significantly reduced p-ERM colocalization with F-actin in stereocilia ( ***Fig. 5B***), decreased the number of p-ERM ^+^ HCs ( ***Fig. 5C***), and resulted in aberrant polarity of stereocilia at the basal turn in *Osbpl2*-WT mouse cochlear explants ( ***Fig. 5D***). In contrast, LPA treatment restored stereociliary p-ERM levels ( ***Fig. 5B***), induced an increase in p-ERM ^+^ HCs ( ***Fig. 5C***, KO+LPA), and partially rescued the aberrant polarity of stereocilia at the basal turn in *Osbpl2*-KO mouse cochlear explants ( ***Fig. 5D***).

## Discussion

OSBP/ORPs constitute a family of intracellular lipid-binding and transport proteins in eukaryotes, playing important roles in lipid metabolism, vesicle transport, and signal transduction ^[ 28– 29] ^. Recent studies have shown that OSBP/ORPs are also involved in cytoskeletal regulation. OSBP functions as a vesicle-associated membrane protein-associated protein interactor, influencing the regulation of phosphatidylinositol 4-phosphate (PI4P) on endosomes and actin nucleation on membranous organelles ^[ 30] ^. Oxysterol-binding protein-related protein 3 (ORP3) regulates the actin cytoskeleton, cell polarity, and cell adhesion by interacting with R-Ras ^[ 31] ^. ORP4 functionally interacts with intermediate filaments, affecting the remodeling of vimentin filaments ^[ 32] ^. ORP8 inhibits the activity of the Wnt signaling pathway, which is essential for the regulation of the actin cytoskeleton and the establishment of cell polarity by inducing ER stress ^[ 33] ^. ORP10 is dynamically associated with microtubules and is involved in intracellular transport ^[ 34] ^. Recent studies have uncovered a novel function of OSBPL2 as a regulator of the actin cytoskeleton in hepatoma cells (Huh7) ^[ 24] ^.

OSBPL2 (ORP2) is the only OSBP/ORP family member identified as being implicated in DFNA, which has attracted growing interest since it was mapped and cloned. OSBPL2 is ubiquitously expressed and exhibits a multitude of functions. Like other OSBP/ORP family members, OSBPL2 shows a high affinity for cholesterol, oxysterols, and phosphoinositides, regulating cholesterol homeostasis and its subcellular distribution in various cell types ^[ 35] ^. OSBPL2 also exhibits distinct functions in specific cellular and tissue contexts. In Huh7 cells, OSBPL2 interacts with F-actin regulators such as DIAPH1, ARHGAP12, SEPT9, and MLC12. RNA-seq of *OSBPL2*-KO hepatocytes revealed the implication of OSBPL2 in actin cytoskeletal regulation through the Rho GTPase signaling pathway, consistent with features such as abnormal F-actin morphology, impaired lamellipodia formation, migration defects, and impaired adhesion and proliferation ^[ 36– 37] ^. Similarly, adhesion capacity was impaired in *Osbpl2*-KO HEI-OC1 cells, with focal adhesion kinase (FAK) signaling being inhibited ^[ 38] ^. Our previous study revealed that OSBPL2 was located at the base of kinocilia/cilia in auditory HCs and supporting cells and functioned in the maintenance of tubulin-based ciliogenesis by regulating the homeostasis of PI(4,5)P _2_ on the cilia membrane ^[ 23] ^. Additionally, OSBPL2 was found to localize in actin-rich HC stereocilia in mouse cochleae ^[ 20] ^, and the pathological changes in HC stereocilia were detected in *OSBPL2*-disrupted pigs ^[ 22] ^. These findings suggest that OSBPL2 may also play an important role in the regulation of the actin cytoskeleton in auditory cells.

Our findings indicate that OSBPL2 functions as a potential effector of the Rho/ROCK2 signaling pathway, affecting the actin cytoskeleton in auditory cells. Rho GTPases function as bridging molecules and molecular switches in intracellular signal transduction and participate in various physiological and biochemical processes such as the regulation of cytoskeleton dynamics, development, proliferation, survival, and regeneration ^[ 39– 41] ^. These Rho GTPases play a critical role in HC development and auditory conduction, with their activation and interaction directly determining the morphological development, electromotility process, and polarity of cochlear HCs. ROCK is one of the key targets of Rho GTPases in actin cytoskeleton reorganization. ROCK1 and ROCK2 are two isoforms that exhibit tissue-specific differential expression and biological functions. ROCK1, which is highly expressed in heart, kidney, skeletal muscle, pancreas, lung, and liver tissues, is implicated in destabilizing the actin cytoskeleton and promoting cell detachment, whereas ROCK2, required for stabilizing the actin cytoskeleton and cell adhesion, is abundantly expressed in brain and lung tissues. A recent study has revealed the important role of ROCK2 in hearing formation and maintenance. Mutations in *PJVK* cause autosomal recessive hearing loss (DFNB59), and Pejvakin modulates actin dynamics to sustain OHC activity and survival in a cell-autonomous manner, which may depend on its interaction with ROCK2 and other Rho effectors ^[ 42] ^. The ROCK2-mediated p-ERM signaling cascade has been shown to modulate noise-induced HC loss by targeting the actin cytoskeleton ^[ 43] ^. Our study demonstrated that ROCK2 was dominantly expressed in mouse brains and cochleae, consistent with previous reports ^[ 43] ^. Notably, *Osbpl2* deficiency results in decreased ROCK2 expression in auditory cells, thereby affecting the downstream signaling cascade.

The ERM proteins, important substrates for ROCK2, have been characterized as adaptor proteins that crosslink the actin cytoskeleton to the plasma membrane. They participate in a wide variety of actin-mediated cellular events, such as microvilli formation, cell adhesion, maintenance of cell shape, cell motility, apoptosis, and membrane trafficking ^[ 44– 45] ^. The three ERM proteins share similar structures, featuring a C-terminal actin-binding domain and an N-terminal FERM-interacting domain. Their activation requires phosphorylation of a conserved threonine residue at the C-terminal. Several kinases have been implicated in regulating ERM protein function through phosphorylation, including ROCK ^[ 46] ^, multiple protein kinase C (PKC) isoforms ^[ 47] ^, p38 mitogen-activated protein kinase (MAPK) ^[ 48] ^, and lymphocyte-oriented kinase (LOK) ^[ 49] ^. However, the specific kinases that directly activate ERM remain to be further investigated in the context of specific tissues or cell types.

It has been found that the p-ERM proteins can serve as cross-linkers between F-actin and the plasma membrane to maintain actin structure and function in cochlear HCs. Noise exposure has been shown to inhibit the Rho/ROCK2 signaling, downregulate p-ERM in mouse cochleae, and induce OHC loss ^[ 43] ^. ERMs integrate Rho GTPase signaling and function as pivotal effectors of Rho GTPases to regulate actin cytoskeletal organization ^[ 50] ^. Among the ERM proteins, radixin is important for auditory development and maintenance, and mutations in radixin ( *RDX*) are associated with DFNB24 in humans ^[ 51] ^. Radixin is predominantly localized along the length of hair cell stereocilia in both the organ of Corti and the vestibular system ^[ 52] ^. *Rdx*-KO mice exhibit hearing loss characterized by early postnatal progressive degeneration of cochlear stereocilia ^[ 53] ^. Similarly, the present study demonstrates that *Osbpl2* deficiency inhibits the Rho/ROCK2 signaling pathway, leading to decreased p-ERM levels in auditory HEI-OC1 cells and reduced p-ERM localization in OHC stereocilia. These changes result in abnormal morphology of the actin cytoskeleton and stereociliary defects, which can be partially rescued by LPA treatment. However, to fully elucidate the regulatory mechanism of OSBPL2 in actin cytoskeleton regulation in auditory cells, further *in vivo* and *in vitro* studies should be carried out through OSBPL2 restoration and RNA interference in the future.

In summary, the present study demonstrates that the OSBPL2-mediated Rho/ROCK2/p-ERM signaling cascade is implicated in the maintenance of the morphology and function of auditory cells by targeting the actin cytoskeleton, providing novel insights into the pathogenesis of *OSBPL2* mutations associated with human DFNA.

## SUPPLEMENTARY DATA

Supplementary data to this article can be found online.
