# Supplementary material for: Osbpl2 deficiency inhibits Rho/ROCK2/p-ERM signaling and impairs actin cytoskeletal regulation in auditory cells
Source: J Biomed Res. 2025 May 20;39(6):574–86. doi: 10.7555/JBR.38.20240389 (PMC12683511; doi:10.7555/JBR.38.20240389)
Supplement: Supplementary file 1 — Supplementary data to this article can be found online. [file jbr-39-6-574-Supplementary.pdf]

Available online at www.jbr-pub.org.cn

Open Access at PubMed Central

JBR

Journal of Biomedical Research, 2025 39(6): 1–5

*Supplementary Data*

# *OSBPL2* deficiency inhibits Rho/ROCK2/p-ERM signaling and impairs actin cytoskeletal regulation in auditory cells

Cheng Zhang[1,△], Qian Yang[1,△], Yajie Lu[1,2], Qinjun Wei[1,2], Rong Zhou[3], Guangqian Xing[4], Xin Cao[1,2], Zhibin Chen[4,✉], Jun Yao[1,2,5,✉]

[1]*Department of Medical Genetics, School of Basic Medical Sciences, Nanjing Medical University, Nanjing, Jiangsu 211166, China;*
[2]*Jiangsu Key Laboratory of Xenotransplantation, Nanjing Medical University, Nanjing, Jiangsu 211166, China;*
[3]*Department of Physiology, School of Basic Medical Sciences, Nanjing Medical University, Nanjing, Jiangsu 211166, China;*
[4]*Department of Otolaryngology, the First Affiliated Hospital of Nanjing Medical University, Nanjing, Jiangsu 210029, China;*
[5]*Department of Otolaryngology-Head and Neck Surgery, the Affiliated Taizhou People's Hospital of Nanjing Medical University, Taizhou School of Clinical Medicine, Nanjing Medical University, Taizhou, Jiangsu 225300, China.*

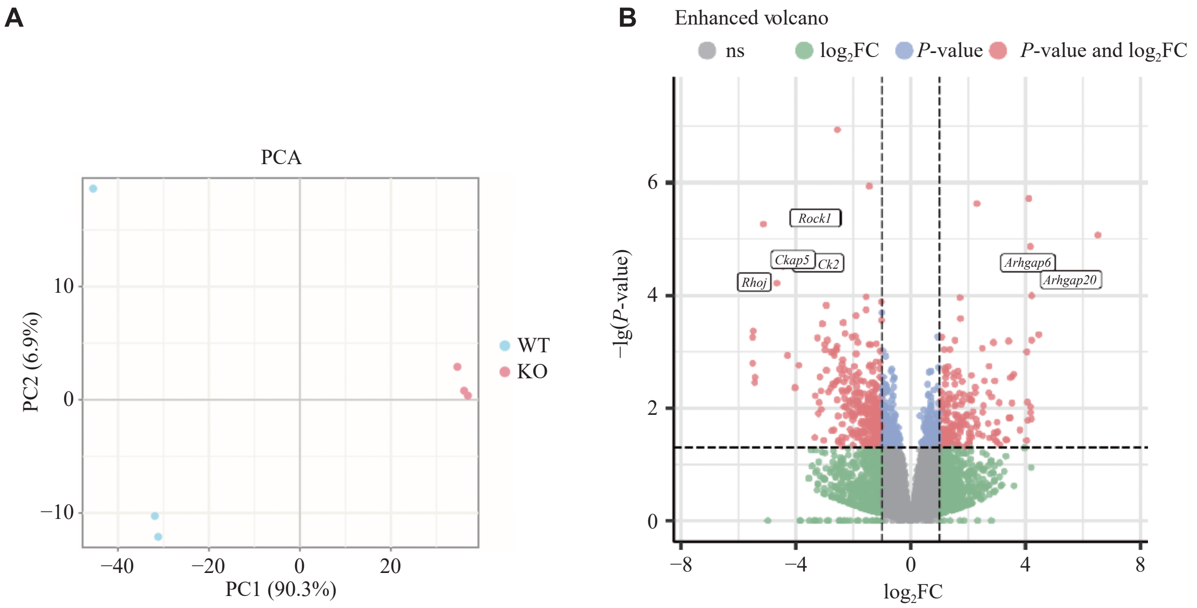


***Supplementary Fig. 1*** **An overall view of RNA-seq profiles in *Osbpl2*-knockout (KO) and wild-type (WT) HEI-OC1 cells.** A: The principal component analysis of RNA-seq profiles in *Osbpl2*-KO and WT HEI-OC1 cells ($n$ = 3). B: Volcano plot of differentially expressed genes in *Osbpl2*-KO and WT HEI-OC1 cells ($n$ = 3). Abbreviations: ns, not significant; FC, fold change.

△These authors contributed equally to this work.

✉Corresponding authors: Jun Yao, Department of Medical Genetics, School of Basic Medical Sciences, Nanjing Medical University, 101 Longmian Avenue, Nanjing, Jiangsu 211166, China. E-mail: joelyao@njmu.edu.cn; Zhibin Chen, Department of Otolaryngology, the First Affiliated Hospital of Nanjing Medical University, 300 Guangzhou Road, Nanjing, Jiangsu 210029, China. E-mail: czbnj@163.com.

Received: 12 November 2024; Revised: 25 April 2025; Accepted: 07 May 2025; Published online: 20 May 2025

CLC number: R764.43, Document code: A

The authors reported no conflict of interests.

This is an open access article under the Creative Commons Attribution (CC BY 4.0) license, which permits others to distribute, remix, adapt and build upon this work, for commercial use, provided the original work is properly cited.

© 2025 by Journal of Biomedical Research.

https://doi.org/10.7555/JBR.38.20240389

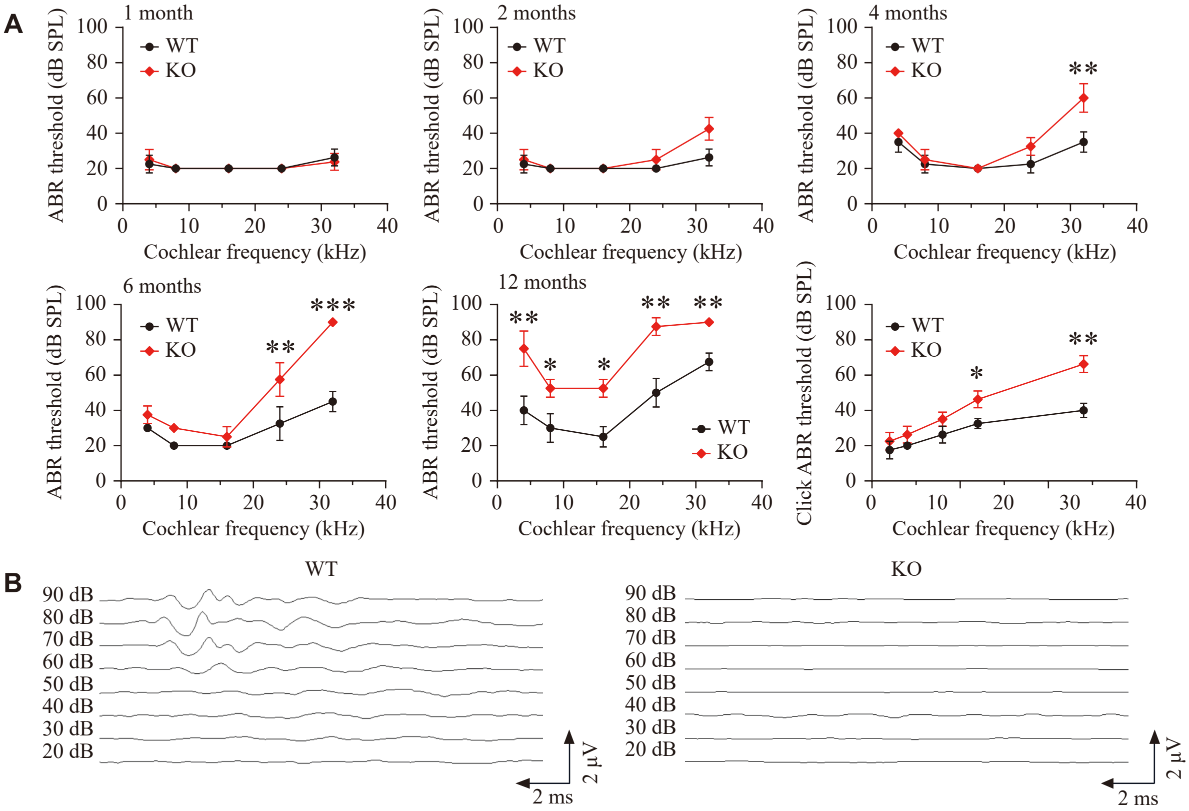


***Supplementary Fig. 2*** **Auditory evaluation in *Osbpl2*-knockout (KO) and wild-type (WT) mice.** The ABR thresholds were assessed at different frequencies in *Osbpl2*-KO and WT mice at the ages of 1, 2, 4, 6, and 12 months. A: Auditory brainstem response (ABR) thresholds of *Osbpl2*-KO mice (red; $n = 6$) and age-matched WT controls (black; $n = 6$). B: ABR waveforms (32 kHz) in 6-month-old *Osbpl2*-KO/WT mice, and the ABR traces were recorded at the same measured range of latency (0–10 ms) and amplitude (0–4 μV). Data are presented as mean and standard error of the mean. $^{*}P < 0.05$, $^{**}P < 0.01$, and $^{***}P < 0.001$ by two-tailed Student's *t*-test. Abbreviation: SPL, sound pressure level.

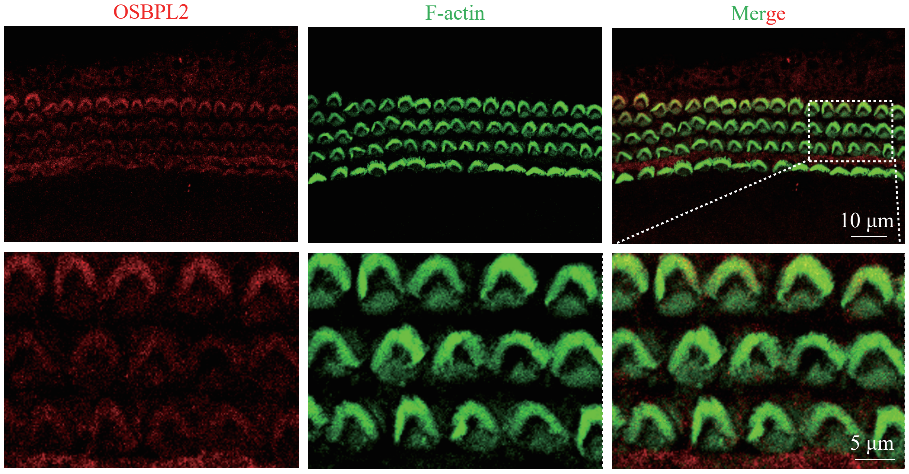


***Supplementary Fig. 3*** **Immunofluorescence staining of the sensory epithelium (basal turn) in 6-month-old wild-type (WT) mice with anti-OSBPL2 (red) and phalloidin (green).** Scale bar: 10 μm. Dashed frames denote the locally zoomed regions as shown below (scale bar: 5 μm). OSBPL2 is co-localized with stereociliary F-actin.

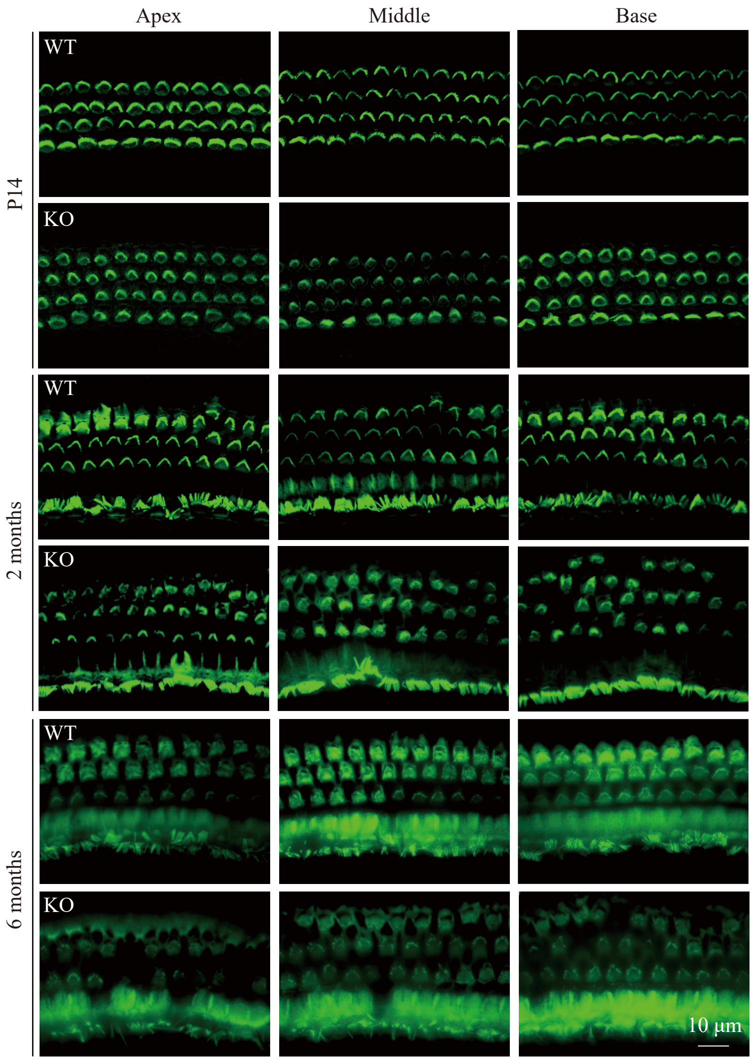


***Supplementary Fig. 4*** **Immunofluorescence staining of the sensory epithelium in *Osbpl2*-knockout (KO) and wild-type (WT) mice at the ages of postnatal day 14, 2 months, and 6 months with phalloidin (green).** Scale bar: 10 μm.

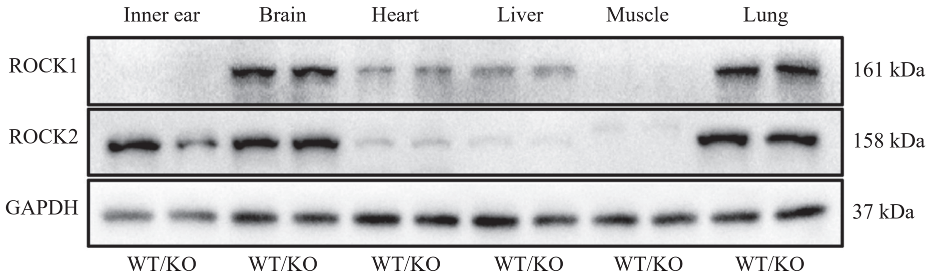


***Supplementary Fig. 5*** **ROCK1/2 expression in mouse tissues.** ROCK2, instead of ROCK1, was dominantly expressed in mouse cochlea. Abbreviation: ROCK1/2, Rho-associated coiled-coil-forming kinases 1/2.

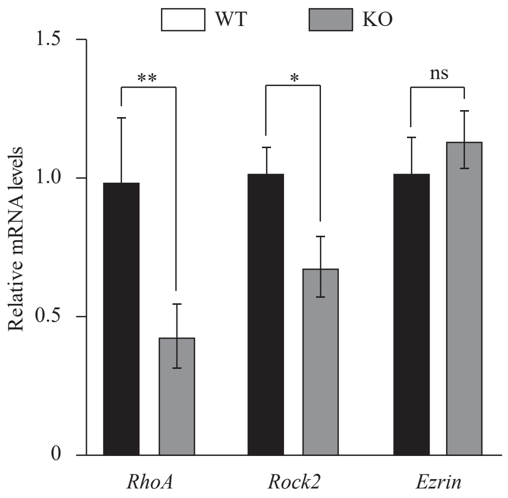


***Supplementary Fig. 6*** **The relative mRNA expression levels of *RhoA*, *Rock2*, and *Ezrin* in cochleae of 6-month-old *Osbpl2*-knockout (KO) and wild-type (WT) mice.** The relative mRNA level of *Ezrin* was used to measure the expression of ezrin-radixin-moesin. Data are presented as mean and standard error of the mean ($n$ = 3). $^{*}P < 0.05$ and $^{**}P < 0.01$ by two-tailed Student's $t$-test. Abbreviation: ns, not significant.

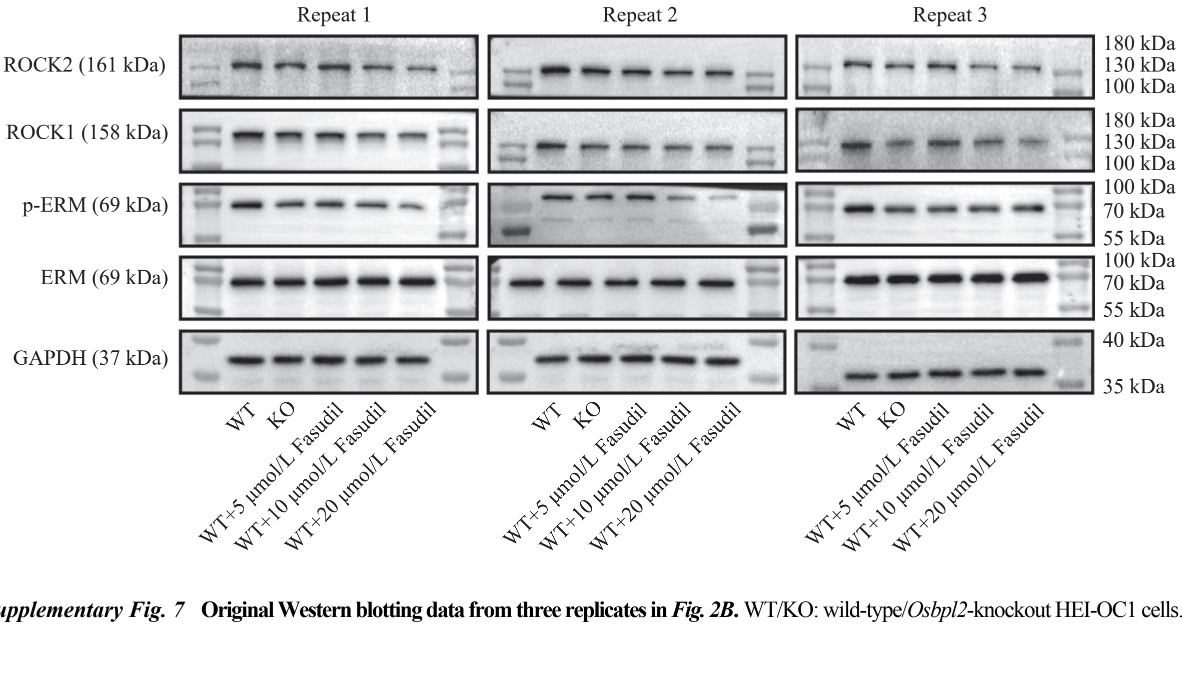


***Supplementary Fig. 7*** **Original Western blotting data from three replicates in *Fig. 2B*.** WT/KO: wild-type/*Osbpl2*-knockout HEI-OC1 cells.

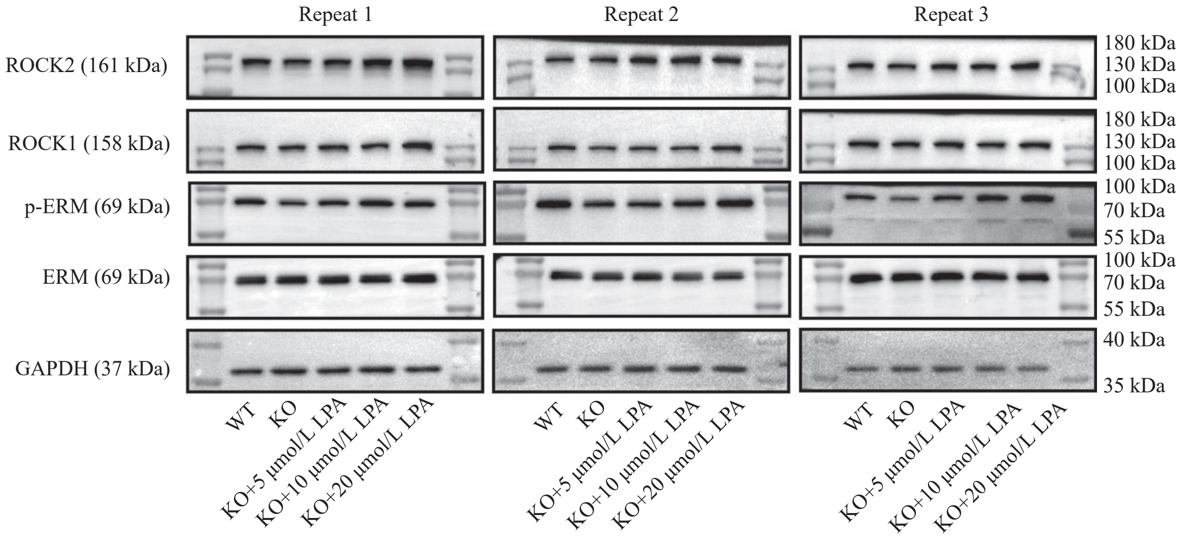


***Supplementary Fig. 8*** **Original Western blotting data from three replicates in *Fig. 2D*.** WT/KO: wild-type/*Osbpl2*-knockout HEI-OC1 cells.

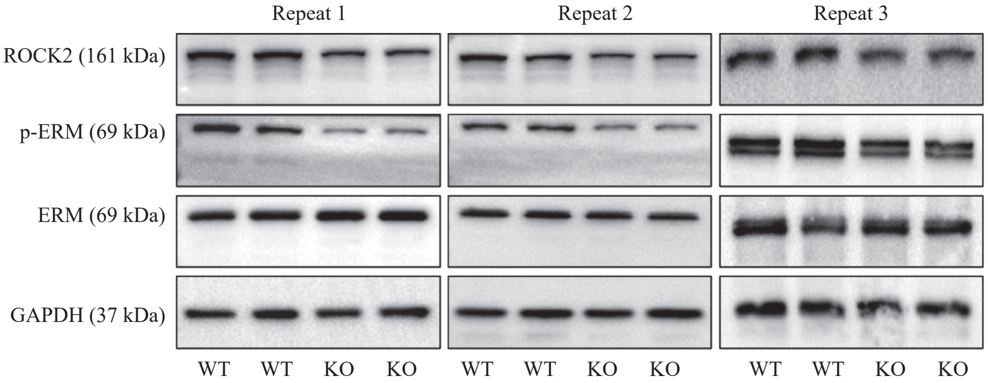


***Supplementary Fig. 9*** **Original Western blotting data from three replicates in *Fig. 4A*.** WT/KO: wild-type/*Osbpl2*-knockout mice.

***Supplementary Table 1*** **Sequences of primers used for qRT-PCR**

| Primers | Sequence (5′-3′) |
|---|---|
| q-M-*RhoA*-F | TGAAGACAGTGAGGGTTTGGG |
| q-M-*RhoA*-R | CATCCACCTCGATATCCGCC |
| q-M-*Rock2*-F | AAACTGTGATCCCAAGGGAAGG |
| q-M-*Rock2*-R | CAACGTCAATCGGAGGCGGA |
| q-M-*Ezrin*-F | CTGGCCCTCATGAAGCAAGA |
| q-M-*Ezrin*-R | TCTGTGGGGGCATGCTTTAG |
| q-M-*Gapdh*-F | AGGTCGGTGTGAACGGATTTG |
| q-M-*Gapdh*-R | TGTAGACCATGTAGTTGAGGTCA |
